# A Rare Case of Ovarian Serous Borderline Tumor with Brain Metastasis

**DOI:** 10.1155/2019/2954373

**Published:** 2019-05-22

**Authors:** Sonia Veran-Taguibao, Roberto Alvaro A. Taguibao, Nicolas Gallegos, Ted Farzaneh, Ronald Kim, Fritz Lin, Di Lu

**Affiliations:** University of California, Irvine Health System, Department of Pathology, Division of Anatomic Pathology, Orange, CA, USA

## Abstract

*Background*. Serous borderline tumor represents a group of noninvasive tumor of the ovary bridging between benign serous cystadenoma and serous carcinoma. They are commonly seen in younger women and usually have an excellent outcome but seldom show local recurrence (J. F. Leake et al. 1991). Metastasis to the lymph nodes has rarely been reported (M. D. Chamberlin et al., 2001; M. B. Verbruggen et al., 2006). Moreover, the brain is exceptionally a rare metastatic site for ovarian tumor. There is one case of an advanced staged SBT with micropapillary pattern metastasis to the brain recently and by far it is the most distant metastasis reported (M. D. Martin et al., 2017). However, to the best of our knowledge, no report has been documented for a recurrent stage 1 typical SBT metastasizing to the brain.

## 1. Introduction

Approximately 3000 American women are diagnosed with borderline ovarian tumors (BOTs) annually and account for 15–20% of all ovarian epithelial tumors. They are known as a tumor of low malignant potential (LMP) or as an atypical proliferative tumor. They are characterized by an increased epithelial proliferation accompanied by nuclear atypia (usually mild to moderate) and low mitotic index in the absence of infiltrative destructive growth or obvious stromal invasion. Histologically, they are subdivided into serous (53.3%), mucinous (42.5%), and the less common, mixed, endometrioid, clear cell, or Brenner tumor (4.2%) [1]. This current case report focuses on serous borderline tumors (SBTs).

SBTs account for one-fourth to one-third of the serous tumors [1, 2]. They occur commonly in the fourth and fifth decades, with an average patient age of 42 years. Although often asymptomatic, the tumor may sometimes present with abdominal enlargement and pain due to rupture or torsion. Approximately 70% of this tumor is confined to one or both ovaries (stage I) at the time of diagnosis. The remaining tumors are found within the pelvis (stage II) or upper abdomen (stage III) and only rare cases have extended beyond the abdomen (stage IV) at the time of presentation [3].

Serous borderline tumor is further subclassified into typical serous borderline tumor, also known as atypical proliferative serous tumor (APST), and the micropapillary variant of serous borderline tumor, which is variably referred to as noninvasive micropapillary serous carcinoma. Typical serous borderline tumor represents approximately three-fourths of the SBTs and is associated with a favorable prognosis, while micropapillary SBT is approximately one-third of the SBT and is associated with unfavorable prognosis.

They are known to spread transperitoneally. Few authors described the spread of this tumor to regional lymph nodes, supradiaphragmatic lymph nodes [4], and internal mammary lymph nodes [5].

It is exceedingly rare to spread to the brain owing to more effective treatment of the primary cancer. However, if it does occur, the most common epithelial ovarian carcinoma associated with this incidence is of serous histotype [6, 7].

## 2. Case Report

**Figure 1:**
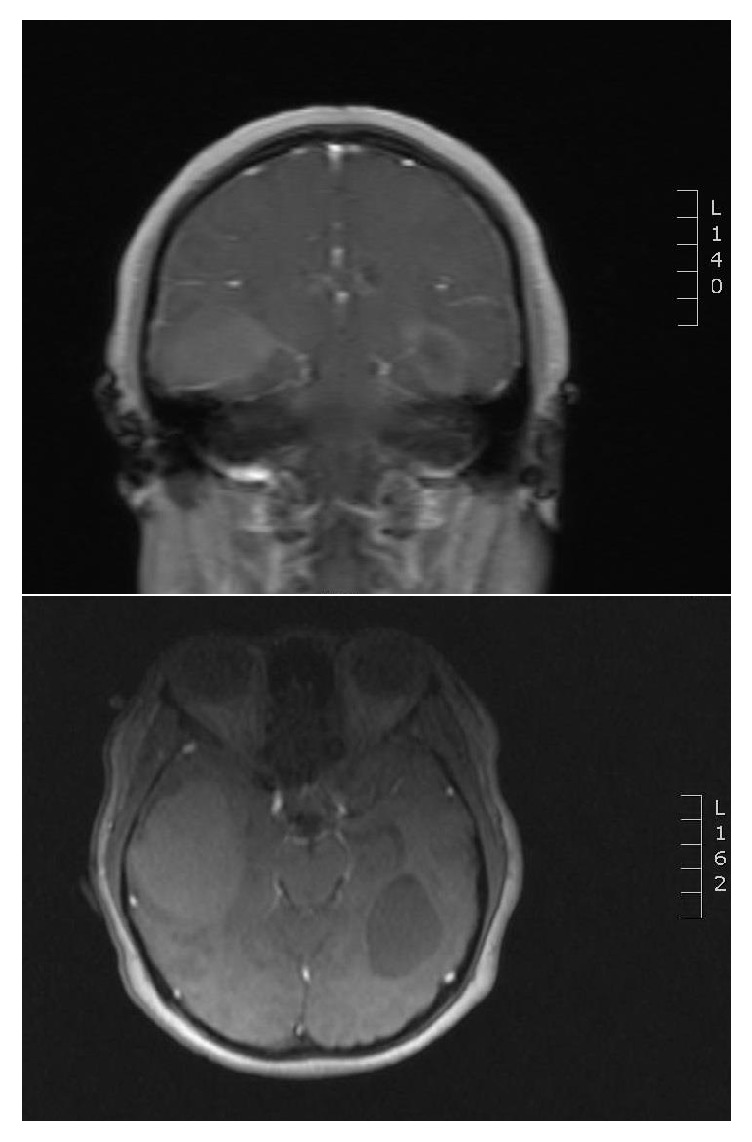
MRI of the brain was done which revealed 4 masses with 2 dominant mass lesions (6.0 and 4.5 cm) having irregular lobulations in the bilateral temporal lobes consistent with metastatic disease.

We present a 33-year-old nulliparous female who presented at our institution with a 3-year progressive headache and was associated with expressive aphasia. MRI of the brain revealed 4 masses including 2 dominant mass lesions (6.0 and 4.5 cm) having irregular lobulations in the bilateral temporal lobes consistent with metastatic disease (Figure 1). Past medical history revealed that unilateral salpingo-oophorectomy with omentectomy, peritoneal washing, and pelvic lymph node samplings were performed twice, 8 and 4 years prior, respectively. Both specimens had serous borderline tumor, one of which had a 1 mm focus of microinvasion.

**Figure 2:**
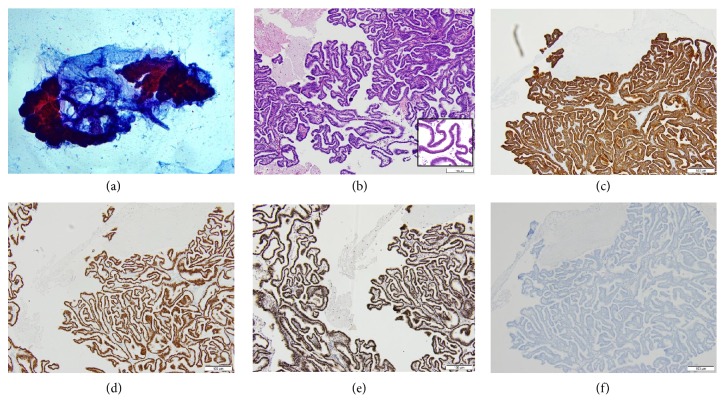
(a) Neoplastic cells forming papillary clusters with smooth contoured edges (b) Serous borderline tumor with hierarchically branched papillae lined by polygonal to columnar serous epithelium with mild atypia (inset) Immunohistochemistry showed (c) CK7 cytoplasmic positivity (d) PAX8 diffuse nuclear positivity (e) WT-1 diffuse nuclear positivity (f) CK20 negative staining.

The fluid sample from the current cystic mass in the brain revealed neoplastic cells forming papillary clusters with smooth contoured edges on the smear (Figure 2(a)). Tissue sample of the brain lesion showed clusters of broad papillae with hierarchical branching and is lined by polygonal to columnar serous epithelium with mild to moderate atypia (Figure 2(b)). Immunohistochemical staining shows positive staining for PAX 8, WT-1, and CK7 and negative staining for CK20 (Figures 2(c)–2(f)). The morphologic features and immunoprofile are in keeping with a diagnosis of the previous ovarian tumor.

## 3. Discussion

The risk factors for SBTs are similar to those for ovarian cancer with notable exceptions of a higher frequency of infertility and a lower frequency of BRCA mutations [8]. The risk factors for recurrent SBTs includes FIGO stage, presence of implants, micropapillary pattern, and microinvasion. Additional factors suggested are incomplete surgical staging, residual disease, fertility-sparing surgery, bilateral ovarian involvement, capsular rupture, and age [9, 10].

Reproductive age group with Stage I SBT, with or without noninvasive implants, can be treated conservatively. According to recent data, recurrence is infrequent ranging from 1.8 to 15%. Therefore, conservative surgery with careful surgical exploration is sufficient [11–13]. In addition, recurrence or development of a new SBT can be effectively treated with reoperation alone for these patients.

Recurrence, though not commonly observed, can occur in a residual ovary especially following fertility-sparing surgery [1, 14]. Preserving the uterus and ovary increases the risk of disease recurrence in the remaining ovary due to the possibility of bilateral synchronous tumors or occult metastases left in situ especially in serous histotype.

Silva et al. observed that, in the recurrences of 11 (6.8%) out of 160 Stage I SBTs treated with total abdominal hysterectomy with bilateral salpingo-oophorectomy, cases showed a higher frequency of endosalpingiosis (72.7%). Thus they suggest that late recurrent tumors could represent new primary serous tumor arising from endosalpingiosis [15]. Another study suggested that glandular inclusions (müllerian cysts) resembling endosalpingiosis seen in the lymph node sample could be a bland appearing form of metastatic SBTs [8, 16].

Although controversial, microinvasion was said to be linked to the recurrence of SBTs. Review of the literatures showed that the overall differences were not statistically significant between typical SBT and SBT with stromal invasion with or without micropapillary features [11]. A retrospective study by Ferrero A et al. compared 209 patients with borderline ovarian tumors (BOT). The microinvasive BOTs had higher recurrence rates (21%) when compared to BOTs without microinvasion (12%), with a median follow-up of 53 months. However, the report also did not yield a statistical significance and the staging for the serous histologic type was not well documented. Seidman JD et al. revealed that stromal microinvasion, if unassociated with extraovarian invasive implants, has no effect on the rate of recurrence or the rate of progression to invasive disease as confirmed in a large meta-analysis [11, 13]. Moreover, overall survival rate for patient with stromal microinvasion that have had stage I disease is at most 91%. Therefore, there is no suggestion for change in the current management [17].

Invasive and noninvasive implants are seen in 35% of the typical SBT patients. However, the invasive implants are known to have higher relapse rate (>50%). Invasive implants are also strongly associated with micropapillary architecture and have worse prognosis than noninvasive implants [1, 9]. If an invasive implant is found in a typical SBT, it suggests insufficient sampling [1] with possible unsampled micropapillary areas or areas of microinvasion. Thus, the current guideline recommends tissue submission of 2 sections per 1 cm for serous histotype tumors for better evaluation of the tumor morphologically [18]. Nonetheless, complete surgical staging and restaging surgery, though controversial, are endorsed for the detection of extra ovarian peritoneal implants which may better assist in the prognosis [1, 19–21].

Other factors associated with recurrence are capsular rupture owing a hazard ratio of 1.9; 95% CI: 1.1-3.6 for recurrent APST and HR of 1.7; 95% CI: 0.4-7.5 for subsequent serous carcinoma [10], and genetic mutations of KRAS and BRAF [22, 23].

Our patient who had previous procedures was clinically stage as FIGO stage I. However, the current procedure in our institution disclosed brain metastasis. A review of the outside pathology reports, except for the 1mm focus of microinvasion, did not show implants or micropapillary pattern, nor capsular rupture. The age of our patient suggested a very early onset ovarian tumor, but next generation sequencing panel for ATM, BARD1, BRCA1, BRCA2, BRIP1, CDH1, CHEK2, EPCAM, MRE11A, MLH1, MSH2, MSH6, MUTYH, NBN, NF1, PALB2, PMS2, PTEN, RAD50, RAD51C, RAD51D, SMARCA4, STK11, and TP53 did not reveal pathogenic mutations, gross deletions, and duplications. No KRAS and BRAF study was done in our patient. She has been treated with brain tumor resection and fractions of intensity-modulated radiotherapy (IMRT) without complications to the resection cavities.

She is followed-up in every 4-month interval and so far, her brain MRI showed no evidence of disease progression. Twenty-three months after her last procedure, she is stable and shows no signs of deterioration.

## 4. Conclusion

To our knowledge, this report represents a rare case of serous borderline tumor metastasizing to the brain. To create a coordinated plan of care for patients with serous borderline tumor, the clinical, surgical, and pathological management of ovarian tumors is of outmost importance in accurate diagnosis and staging of the tumor.
